# Renal-limited AL amyloidosis – a diagnostic and management dilemma

**DOI:** 10.1186/s12882-018-1118-8

**Published:** 2018-11-06

**Authors:** Kar Wah Fuah, Christopher Thiam Seong Lim

**Affiliations:** 1 Department of Medicine, Hospital Tengku Ampuan Afzan, Kuantan, Malaysia; 2 Unit of Nephrology, Department of Medicine, Faculty of Medicine and Health Sciences, Universiti Putra Malaysia, 43400 Serdang, Malaysia

**Keywords:** Localized amyloidosis, Nephrotic syndrome, Amyloidosis

## Abstract

**Background:**

Amyloidosis is a disorder caused by extracellular tissue deposition of insoluble fibrils which may result in a wide spectrum of symptoms depending upon their types, sites and amount of deposition. Amyloidosis can be divided into either systemic or localized disease.

**Case presentation:**

We present a case of a middle-aged gentleman who presented with persistent nephrotic syndrome with worsening renal function. Repeated renal biopsies showed the presence of renal-limited AL amyloidosis. Systemic amyloidosis workup was unremarkable apart from a slightly raised band of IgG lambda level with no associated immunoparesis. The nephrotic syndrome and renal histology did not improve over a 3-year period despite being given two courses of chemotherapies.

**Conclusion:**

We hope that early recognition of this unusual localised presentation of renal- limited AL Amyloidosis and its poor response to conventional treatment can alert the nephrologist to the potential existence of this rare condition.

## Background

Amyloidosis are characterized by insoluble fibrils from a variety of low molecular weight subunits with their characteristic appearance on the electron microscopy and by their virtue of yielding green birefringence under polarized light in the Congo red staining [1]. The four commonest types of amyloids are immunoglobulin light chain (AL), amyloid A (AA), transthyretin (ATTR) and amyloid Beta peptide (AB) [2]. Systemic amyloidosis is further categorized into primary and secondary amyloidosis, which carries the risk of progression into neoplastic disease. Systemic amyloidosis commonly affects tissues such as kidneys, cardiac, peripheral nerves and musculoskeletal tissues. On the other hand, localized amyloidosis is rare and has a different character as compared to systemic amyloidosis. In fact, localized amyloidosis has never been known to progress systemically. Localized amyloidosis is commonly found in upper airway (nasopharynx, tongue), orbits, urinary tract including urinary bladder and musculoskeletal tissues (skin and nails).

## Case presentation

A 53-year-old, non-diabetic, Chinese gentleman, presented with multiple episodes of bilateral lower extremities edema in 2012. There was no prior history of weight loss, skin rash, heart failure symptom or numbness over the lower extremities. Physical examination revealed a well-built gentleman with an elevated blood pressure reading of 160/90 mmHg. There was bilateral lower limbs edema but no organ enlargement was noted. Blood investigation showed normal full blood count, mildly impaired renal function with serum creatinine of 202 μmol/L and hypoalbuminemia without raised globulin level. Connective tissue disease screening was negative. 24 h urinary protein collection revealed proteinuria of 9 g per day Ultrasonography scan of the kidneys showed normal renal parenchymal echogenicity with bilateral kidneys’ size measuring at 9.5 cm and 9.6 cm respectively. Renal biopsy carried out showed AL amyloidosis with no evidence of free light chain deposition (as evidenced by the Congo red staining and negative for other specific staining). Extensive workup to look for other features of primary amyloidosis failed to show any association with systemic involvement (bone marrow aspiration and trephine biopsy, skeletal survey, echocardiogram, rectal biopsy was all reported negative). The only positive results was from the serum electrophoresis whereby it demonstrated the presence of IgG lambda paraprotein < 0.2 g/L migrating towards beta zone without any evidence of immunoparesis. The urine electrophoresis showed albuminuria of 14.1 g/L with mixed IgG lambda paraproteinuria of < 0.15 g/L. He was subsequently referred to hematologist for an opinion and was treated with 2 cycles of CTD (cyclophosphamide, thalidomide and dexamethasone) and VTD (bortezomide, thalidomide and dexamethaosone) with no resolution of the nephrotic syndrome. A repeated renal biopsy performed 3 years later showed no histological difference as compare with the first biopsy. A repeated systemic amyloidosis workup again showed inconclusive result. Bone marrow aspiration and biopsy repeated showed normal cellularity with presence of 2–3% plasma cells likely to be reactive in nature. Flow cytometry result showed 6% lymphocytes and 0.5% plasma cells with no aberrant plasma cells detected. He remains in overt proteinuria (7.8 g–10 g/dl) with a slowly creeping serum creatinine.

## Discussion and conclusion

AL amyloidosis is a monoclonal plasma cells disorder which has a variety of symptoms based on the system it is affecting. It’s formerly known as primary amyloidosis in which it tends to behave in a malignant manner with metastatic property but yet remains to be chemo-responsive. It’s not uncommon to find one system involve more prominently over the others in AL amyloidosis. The common sites of involvement include kidney, liver, cardiac, peripheral neuropathy, musculoskeletal and skin. Amyloidosis has a strong association with other plasma cell dyscrasias, namely multiple myeloma, monoclonal gammopathy undetermined significance (MGUS) or Waldenstrom macroglobulinemia [3].

In systemic amyloidosis, the plasma cell clones secrete monoclonal immunoglobulin (Ig) light chain (LC) which is then deposited on the specific organ. Immunoglobulins light chains are further identified into lambda or kappa light chains. Localization of amyloid tissue to one particular organ is very common, particularly in association with the aging process. Common examples include amyloid deposition in the brain tissues in Alzheimer’s disease and in islets of Langerhans in type 2 diabetes. These tend to appear as multiple deposits. On the other hand, in localized AL amyloidosis, it often appears as one, often tumor-like or pseudotumor lesion, although multiple nodules may occur. Localized AL amyloidosis produces misfolded protein precursor (immunoglobulin light chain) which is then deposited locally, causing obstruction via its mass effect. Localized AL amyloidosis is most commonly found in upper airway (nasopharynx, tongue), orbits, urinary tract including urinary bladder and musculoskeletal (skin and nails) [4–7]. The natural course of the disease is relatively benign in most patients, but severe damage to the affected organ may occur.

Kidney as the sole site of localized AL amyloidosis manifestation has never been reported in the literature before. Postulation of the pathogenesis of renal amyloidosis is mainly derived from systemic amyloidosis. The immunoglobulins light chains typically affect the glomerular and tubules. Hence, renal amyloidosis usually presents with significant degrees of proteinuria, legs edema and renal impairment [8, 9]. However, in a small proportion of patients, renal impairment without significant proteinuria is the presenting features [10]. It has been postulated that, apart from the systemic deposition of the light chain, the mesangial cells in the nephron can undergone mutation and produce amyloid fibrils [11]. Theoretically speaking therefore, localized AL renal amyloidosis is not a myth.

**Fig. 1:**
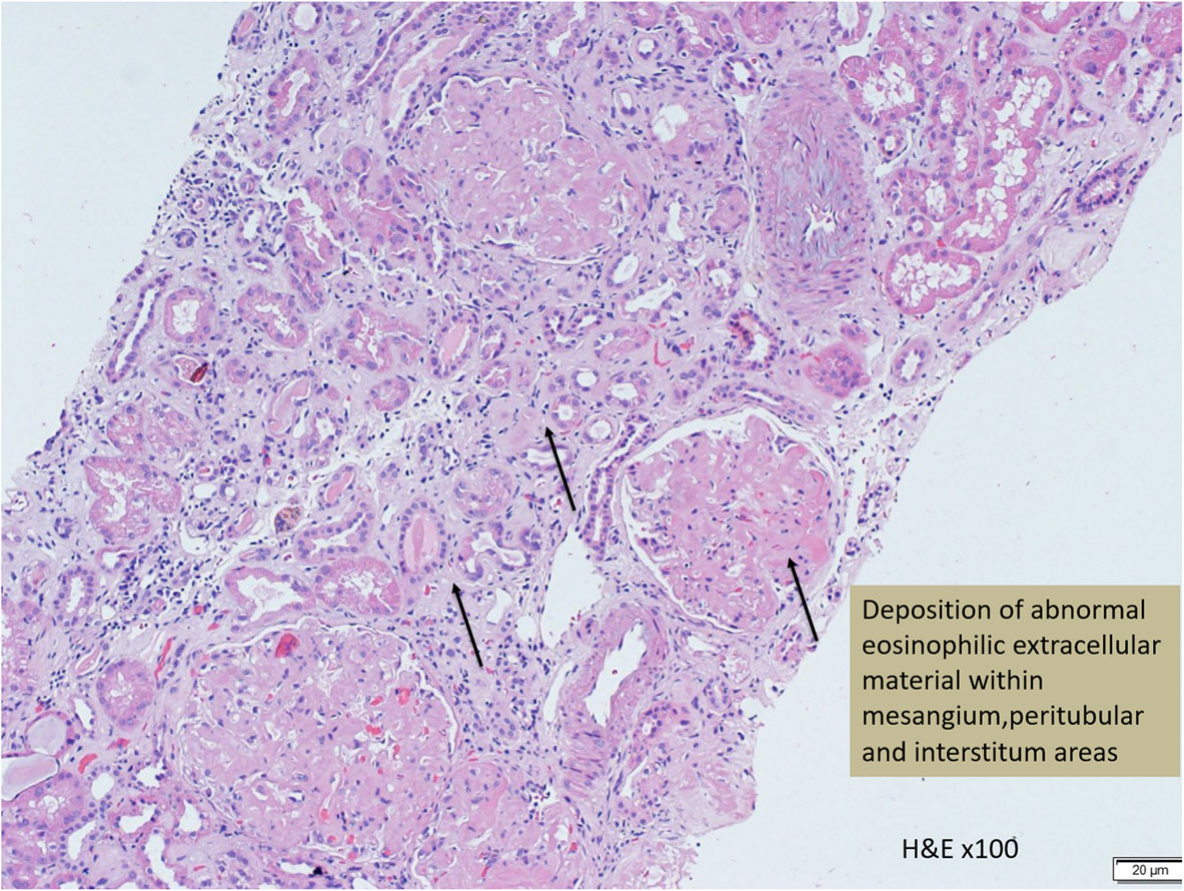
(H&E × 100): Deposition of abnormal eosinophilic extracellular material within mesangial, peritubular and interstitial

**Fig. 2:**
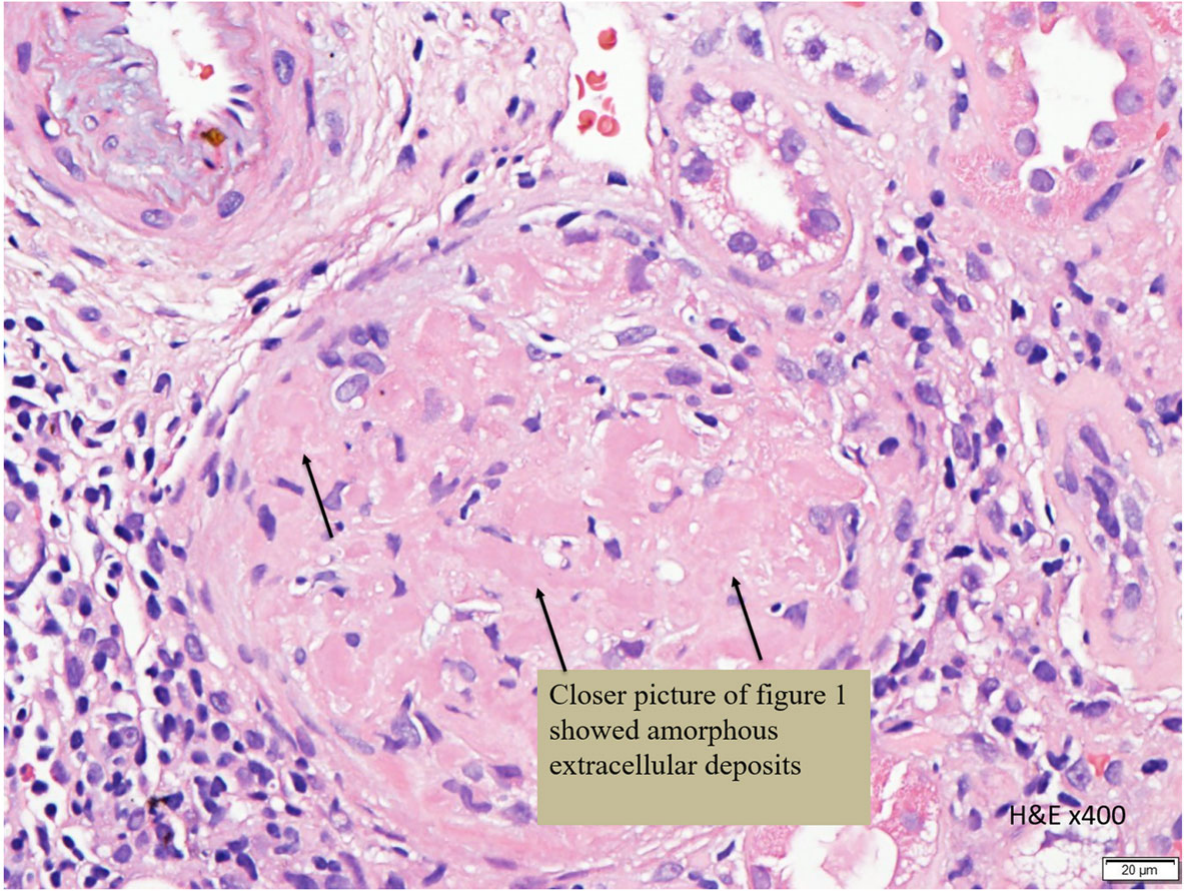
(H&E × 400): Closer picture of Fig. 1 showed amorphous extracellular deposits

**Fig. 3:**
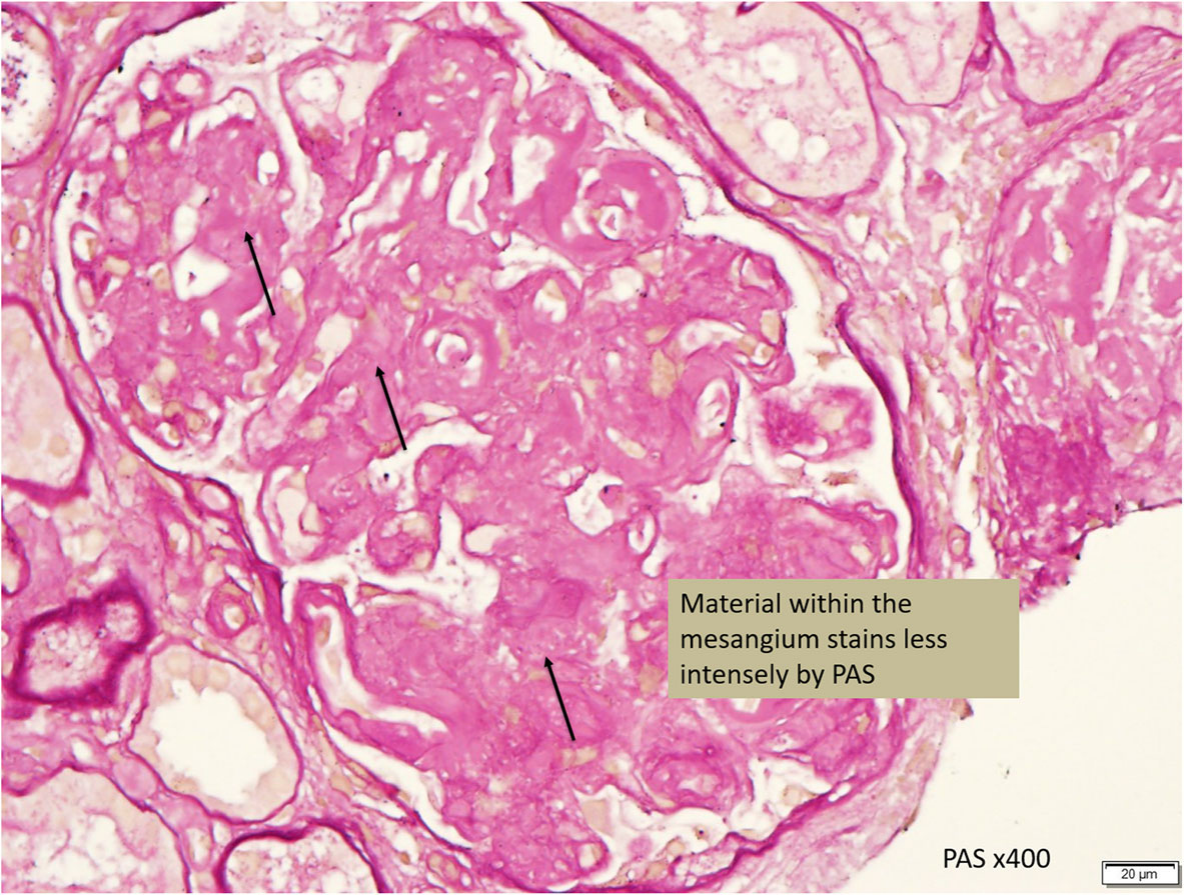
(PAS × 400): Material within the mesangial stains less intense

**Fig. 4:**
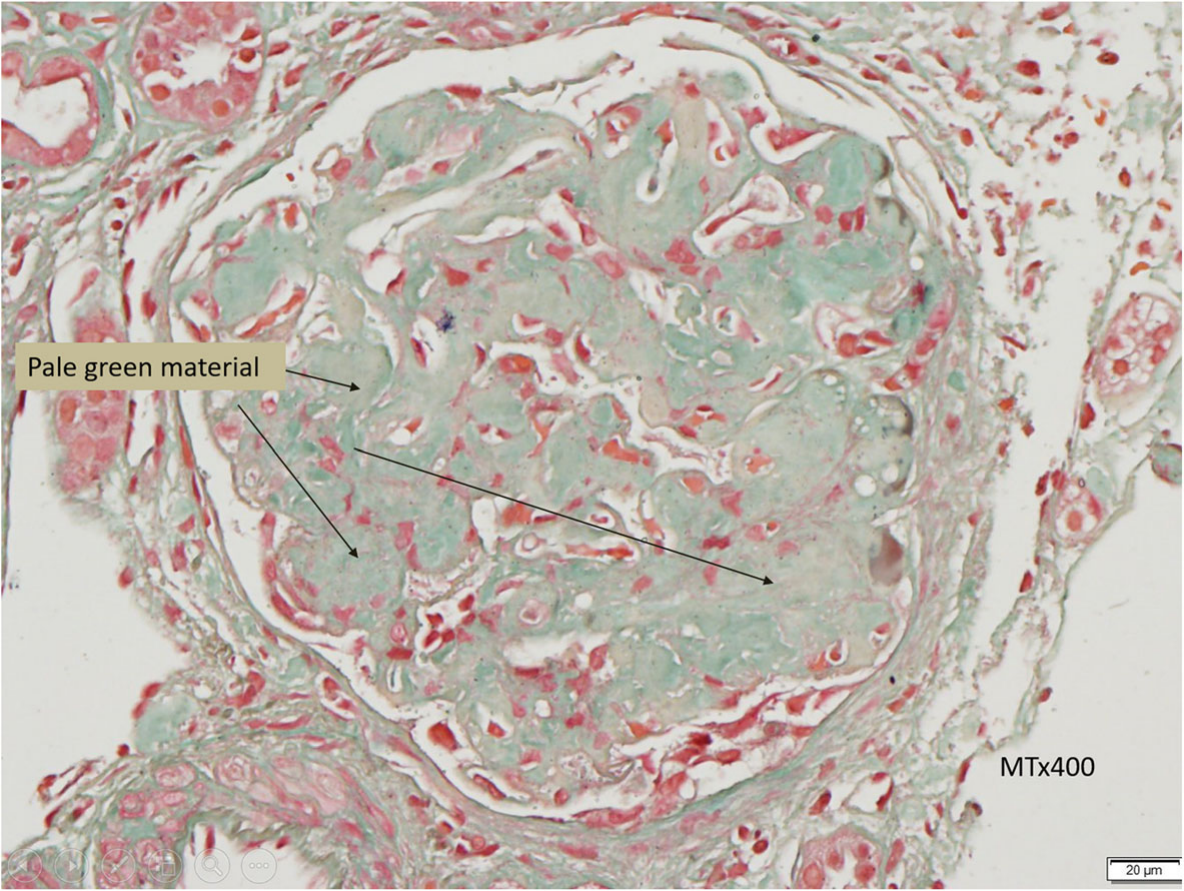
(MT × 400): Pale green materials at the interstitial

**Fig. 5:**
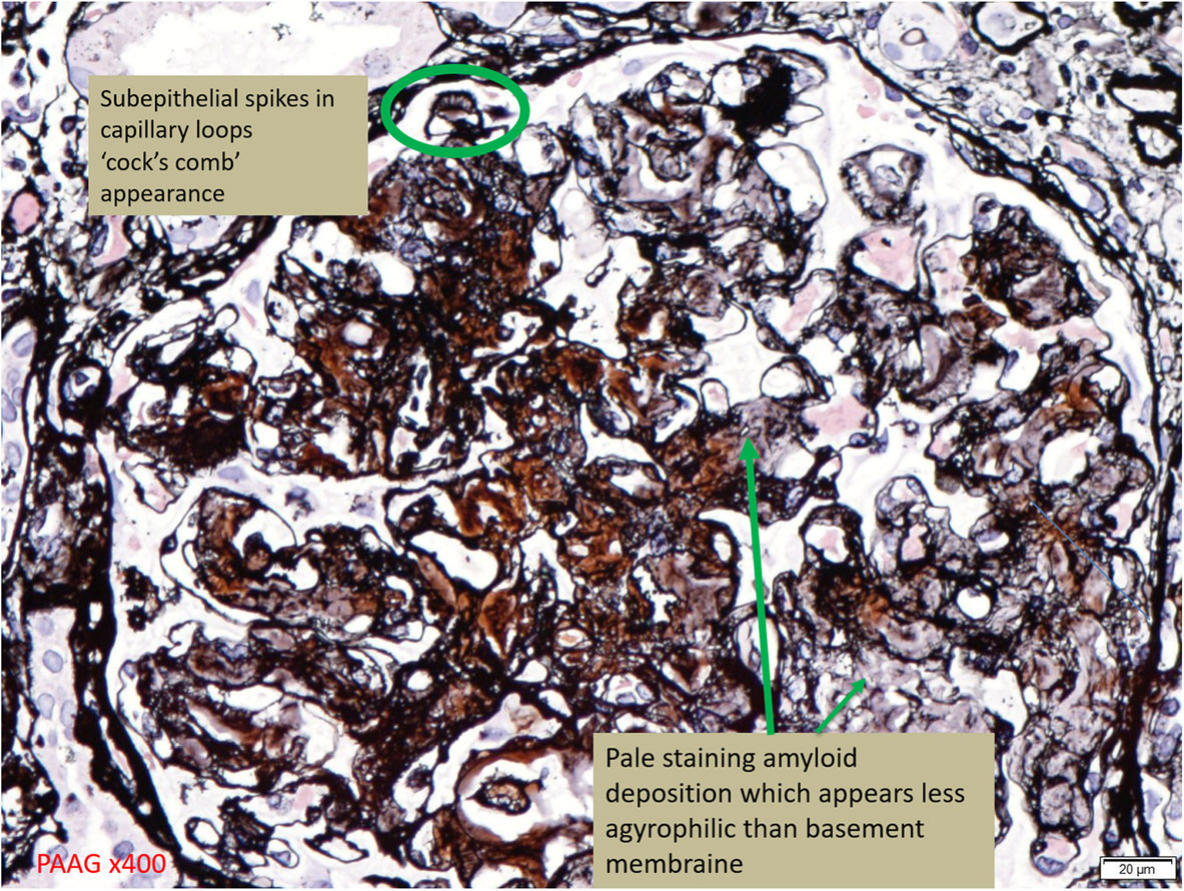
(PAAG x 400): Subepithelial spikes in capillary loops with ‘cock’s comb’ appearance

**Fig. 6:**
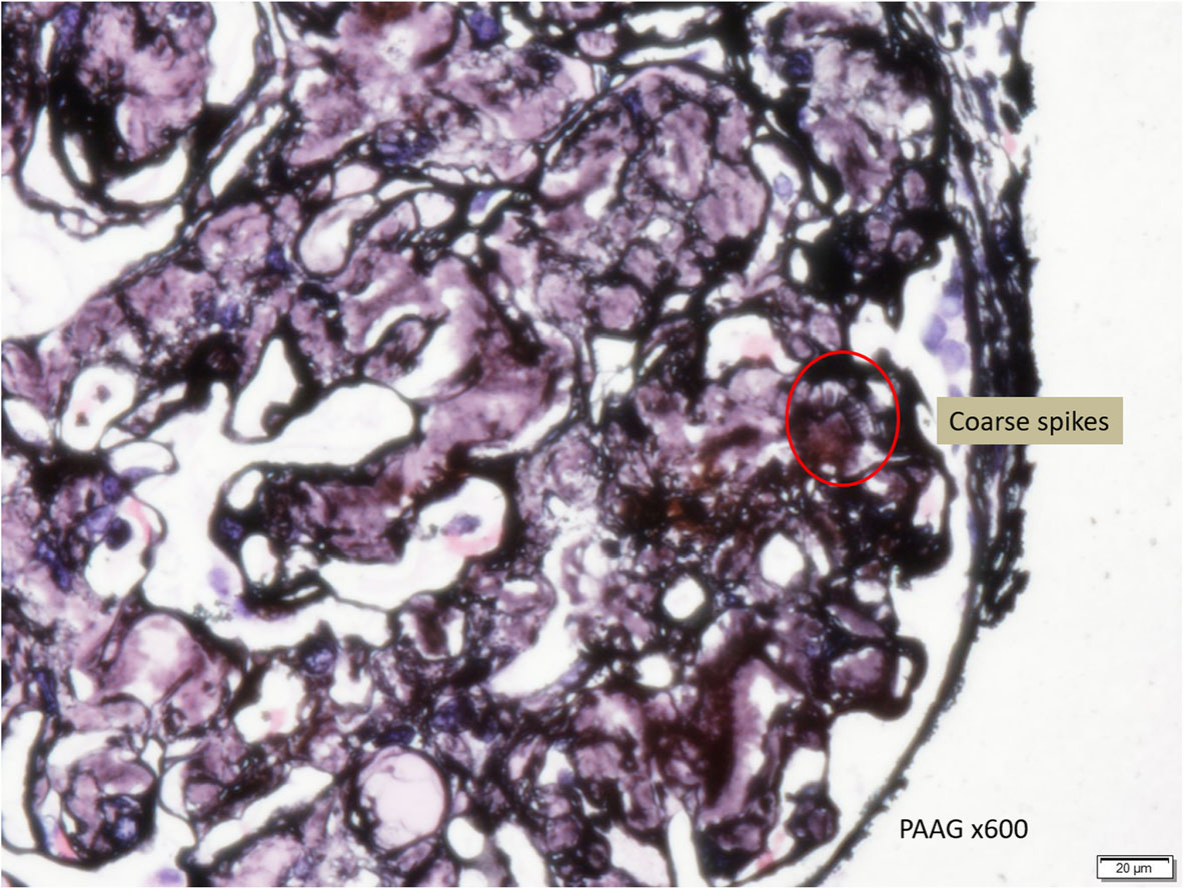
(PAAG x 600): Subepithelial coarse spikes

**Fig. 7:**
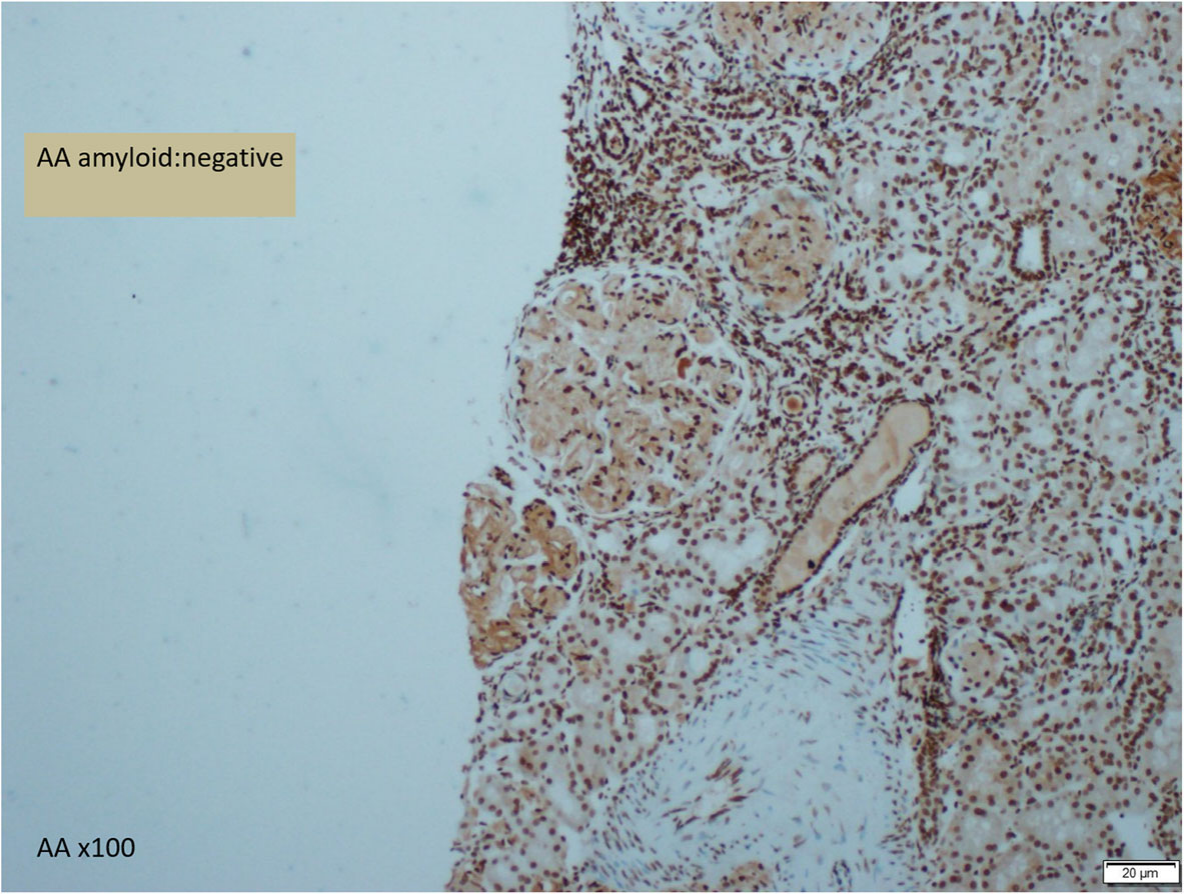
(AA x 100): AA amyloid stain is negative

In this case, a non-diabetic patient with massive proteinuria, mild renal impairment, normal globulin/albumin ratio raised the initial clinical suspicion of possible amyloidosis. Hence a renal biopsy became mandatory. Under Hemotoxylin & Eosin (H & E) stain, amyloid appears salmon pink, amorphous extracellular deposition (Figs. 1, 2). staining in this case showed no thickening of the basement membrane and the depositions were faintly stained (Fig. 3). Masson’s Trichrome (MT) stained the extracellular depositions pale green in colour (Fig. 4). Periodic acid-silver methenamine (PAAG) silver stain showed the amyloid deposits as subepithelial spikes in capillary loop and the deposits were stained less argyrophilically than the adjacent basement membrane (Figs. 5, 6). Congo red staining revealed the typical faint apple- green birefringence under polarized light source. Further immunofluorescent staining was performed to identify the type of the amyloids. In AA amyloidosis, complement and immunoglobulin will be absent whereas light chain (kappa/lambda) will be present in AL amyloids. In our patient, there was nonspecific staining of C3 with negative staining for heavy chains, light chains and C1q (Fig. 7). Interaction between amyloid and immunofluorescent reagent sometimes can give false positive result especially in AA amyloid [12]. On the other hand, false negative result for immunoglobulin light chain does occur with a rate of 25% due to technical issues. When the classification of amyloidosis is in doubt, laser dissection with mass spectrometry -based proteomic analysis should be performed [13]. However, this is not done due to budget constraint at the local facility. In our case, an extra step of potassium permanganate treatment prior to Congo red staining was done and this yielded persistent birefringence which suggested AL amyloid instead of AA. All the renal stains above were repeated twice in our case.

Similarly, serum and urine protein electrophoresis carried out did not show convincing pattern of MGUS to support for presence of systemic AL amyloidosis. MGUS is an asymptomatic pre-malignant plasma cell disorder. It is typically an incidental finding of serum paraprotein < 3 g/dL, bone marrow plasma cells< 10% with absence of end organ damage or mass effect due to the lymphoproliferative process [14]. The presence of renal involvement in our case made MGUS as an unlikely cause of the nephrotic syndrome.

It is worthwhile to note that the management for systemic AL amyloidosis, localized amyloidosis and MGUS are distinctively different. Treatment for systemic amyloidosis mainly involves administering chemotherapy to suppress the plasma cell clonal expansion. In localized amyloidosis, treatment is generally confined to local surgical intervention and resection. Whilst in MGUS, due to its pre-neoplastic nature with an annual rate of 1% progression into advance disease, a close periodic monitoring is mandatory to detect its progression and its related complications [15, 16]. In our case, despite this a is localised amyloidosis, a trial of 2 full courses of chemotherapies were given with no meaningful renal response observed. Until to date, the patient is still experiencing massive proteinuria with slowly worsening of renal function. Unfortunately, there is no other routine markers that we can use to monitor the progression of renal limited AL amyloidosis. In our opinion, it is prudent to meticulously follow up the patient, employ various strategies to retard the proteinuria with careful monitoring of the renal function and paraprotein level.

In conclusion, we reported the first case of renal limited amyloidosis secondary to AL amyloidosis. We hope that early recognition of this unusual localised presentation of a AL amyloidosis and its poor response to conventional treatment can alert the nephrologist to the potential existence of this challenging condition. A faithful monitoring of the patient’ serum plasma paraprotein status and effective proteinuria reduction should be incorporated as the main stay of treatment.

## Abbreviations

AA: Amyloid A
AB: Amyloid Beta peptide
AL: Amyloid immunoglobulin light chain
ATTR: Amyloid transthyretin
CTD: Cyclophosphamide, thalidomide and dexamethasone
H & E: Hemotoxylin & Eosin
Ig: Immunoglobulin
LC: Light chain
MGUS: Monoclonal gammopathy of undetermined significance
MT: Masson’s Trichrome
PAAG: Periodic acid-silver methenamine
PAS: Periodic Acid Schiff
VTD: bortezomide, thalidomide and dexamethaosone
